# A Patient-Prioritized Agenda for Information Needs During the COVID-19 Pandemic: A Qualitative Study of Patients With Inflammatory Bowel Disease

**DOI:** 10.1093/crocol/otab066

**Published:** 2021-09-19

**Authors:** Millie D Long, Mary E Grewe, Emily Cerciello, Laura Weisbein, Kyra Catabay, Michael D Kappelman

**Affiliations:** 1 University of North Carolina, Department of Medicine, Division of Gastroenterology and Hepatology, Chapel Hill, North Carolina, USA; 2 Center for Gastrointestinal Biology and Disease, University of North Carolina, Chapel Hill, North Carolina, USA; 3 University of North Carolina, NC TraCS Institute—Community and Stakeholder Engagement Program (CaSE), Chapel Hill, North Carolina, USA; 4 Crohn’s and Colitis Foundation, New York, New York, USA; 5 University of North Carolina, Department of Pediatrics, Division of Gastroenterology and Hepatology, Chapel Hill, North Carolina, USA

**Keywords:** inflammatory bowel disease, Crohn’s disease, ulcerative colitis, COVID-19, focus groups

## Abstract

**Background:**

Patients with inflammatory bowel disease (IBD) may be at risk for complications due to the COVID-19 pandemic. We performed a qualitative study to better understand IBD patient experiences and concerns when navigating the COVID-19 pandemic, with the goal of prioritizing patients’ information needs.

**Methods:**

We conducted a series of semistructured virtual focus groups at 6 months, then member checking focus groups 1 year into the COVID-19 pandemic. We included questions on patients’ experiences navigating the pandemic with IBD, differences in their experience as compared to peers, their concerns and fears, as well as preferred information sources. Transcribed focus groups were coded and content analyzed to summarize key areas of interest and identify themes. We focused on 4 areas in our content analysis process: fears, challenges, information preferences, and research questions.

**Results:**

A total of 26 IBD patient participants were included in the initial focus groups. Findings highlighted the many challenges faced by patients during the COVID-19 pandemic, ranging from access (bathrooms, medications, healthcare) to significant fears and concerns surrounding medications used for IBD worsening risks of COVID-19. Research questions of importance to patients centered on understanding risks for COVID-19 complications, particularly pertaining to medication utilization, with a shift over time toward understanding COVID-19 vaccination. In our member checking focus groups (*n* = 8 participants), themes were reiterated, with a central focus of research questions pertaining to COVID-19 vaccination.

**Conclusions:**

Information needs for patients during the COVID-19 pandemic centered upon understanding disease-specific risks. Identified challenges and fears will inform future research agendas and communication with patients.

## Lay Summary

We used focus groups to understand the concerns of patients with inflammatory bowel disease (IBD) during the COVID-19 pandemic. Patients’ fears were linked to medications. Patients’ questions shifted from understanding the risks of IBD medications to understanding COVID-19 vaccination.

## Graphical Abstract

**Figure:**
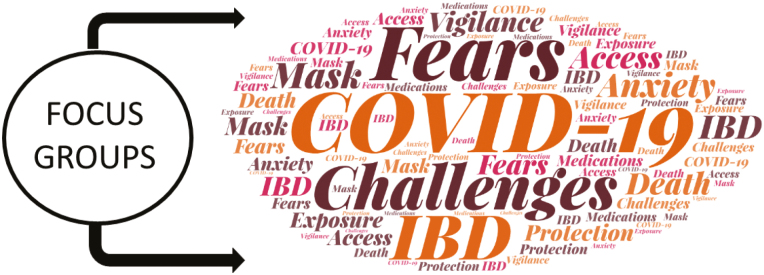

## Introduction

Over the course of the COVID-19 pandemic, there has been a high level of patient demand for information about COVID-19 and its impacts on the inflammatory bowel disease (IBD) community. The Crohn’s & Colitis Foundation is a nonprofit organization with a mission to find cures for Crohn’s disease and ulcerative colitis and to improve the quality of life of children and adults affected by these diseases. As the major advocacy organization for the IBD community, the foundation serves as an information source for hundreds of thousands of patients with IBD. In 2020 alone, the Crohn’s & Colitis Foundation had over 1 million engagements with their COVID-19 resources.^1^ Early data during the pandemic described IBD patient experiences with COVID-19 infection from various countries around the world.^2–5^ The international SECURE-IBD registry^6^ provided further information via investigation of medication-specific risks of developing complications from SARS-CoV-2 infection. Many patients contacted providers and advocacy organizations to better understand IBD-specific risks and outcomes of SARS-CoV-2 infection in the setting of limited available data.

With a rapidly changing information flow to patients during the COVID-19 pandemic, qualitative research became imperative to better understand the perspectives of individuals with IBD. Focus groups are 1 mainstay of qualitative research. Focus groups, a form of group interview, capitalize on communication between research participants in order to generate data. This method is particularly useful for exploring people’s knowledge and experiences, to better understand not only how participants think, but also why they think that way.^7^ This methodology is therefore appropriate to understand the perspectives of the IBD community over the course of the COVID-19 pandemic.

To better inform patient communication, educational materials, and research priorities, we aimed to use a series of qualitative focus groups to (1) better understand the experiences of IBD patients during the COVID-19 pandemic and (2) prioritize patients’ information needs (ie, research questions) and preferences for making informed decisions (recognizing the trade-off between time and quality of data). Additionally, as the pandemic progressed, we aimed to see how these priorities changed over time.

## Methods

### Setting and Participants

Our target population was individuals with IBD. Recruitment was conducted via social media efforts of the Crohn’s & Colitis Foundation, including recruitment messages on Facebook, Twitter, at chapter meetings and through email. Eligibility criteria included age 18–99 years; self-reported IBD diagnosis, English language, and access to a computer with a video camera for the virtual focus groups. Those patients with confirmed eligibility were invited to participate in a virtual focus group via Zoom. Verbal informed consent was obtained, and a modest financial incentive was provided.

### Data Collection and Analysis—Initial Focus Groups

Between August and October of 2020, a total of five 90–120-minute focus groups were conducted. Number of participants in each focus group ranged from 3 to 5. The focus group guide was developed based on input from clinicians, researchers, and our advocacy organization for IBD, the Crohn’s & Colitis Foundation (Table 1). Participants were asked about experiences navigating the COVID-19 pandemic as someone living with IBD, differences in their experience as compared to peers, their concerns and fears, preferred information sources, and priority research questions.

**Table 1.** Round 1 focus group questions

Participants were asked to describe their experience navigating the COVID-19 pandemic with IBD	
What has been your experience navigating the COVID-19 pandemic as someone living with IBD?	What questions related to IBD and COVID-19 do you think researchers should focus on?
How is your experience different than that of family and friends without IBD?	How are you getting information about COVID-19?
What concerns or fears do you have about COVID-19?	Would you rather hear new information right away, or wait for more accurate information?

Abbreviation: IBD, inflammatory bowel disease.

Focus group recordings were professionally transcribed and confirmed by a study team member. Two team members (M.E.G. and E.C.) coded the focus group transcripts. Each team member independently coded the first transcript, then reviewed and discuss codes until consensus was reached. The initial codebook was developed based on the codes derived from the initial transcript. They repeated this process for the second transcript. Once this process was complete, the codebook was stable. One team member (M.G.) then coded the remaining 3 transcripts, which the other team member (E.C.) reviewed. Any disagreement in coding was discussed until consensus was reached. In addition, summary memos for each focus group were generated to highlight key concepts discussed within each session. Coding was managed in Dedoose.

We further content analyzed the data for the following codes of key interest: fears, challenges, research questions, information preferences, and trustworthy sources, developing summary reports that organized content topically within these areas of interest, and allowed us to identify overall themes within the data.

### Member Checking Focus Groups

In April 2021, we conducted 2 additional 90–120-minute member checking focus groups. Number of participants in each focus group ranged from 3 to 5. During these focus groups, we shared findings from the initial focus group, and asked participants to react. In particular, we asked them to share whether the findings reflected their experience and priorities and how these might have changed over time. In addition, we asked them to share their perspectives and information preferences related to COVID-19 vaccines. Focus group recordings were professionally transcribed. A study team member reviewed the transcripts and developed memos summarizing the feedback obtained during the sessions and including illustrative quotes. The focus group facilitator (M.G.) reviewed these memos, providing feedback and additional information as needed.

## Results

A total of 26 participants participated in 5 focus groups in the initial study period, and 8 participants participated in the member checking focus groups 1 year into the pandemic. We categorized findings related to the following areas of focus: fears, challenges navigating the COVID-19 pandemic as someone living with IBD, information preferences, and prioritized research questions (Table 2).

**Table 2.** Themes and examples from focus groups of prioritized information needs for patients with IBD

Theme	Examples
Fears during COVID-19 pandemic	Exposure to COVID-19 when accessing healthcare
	Public restrooms
	Increased stress causing flares
	Medications impacting risks of COVID-19
	Access to COVID-19 vaccine
	Safety of COVID-19 vaccine
Challenges navigating the COVID-19 pandemic as someone living with IBD	Accessing medications, treatment delays
	Issues navigating bathroom access
	Uncertainty about whether someone with IBD is at high risk
	Accessing healthcare providers/medical care
	Needing to go to appointments alone
	Effects on family and friends trying to protect a high risk patient with IBD
	Concerns of others not taking the pandemic seriously and putting IBD patients at risk
	Exposure to COVID-19 in everyday life
Information preferences (trustworthy sources)	Crohn’s and Colitis Foundation
	Government organizations
	Patient communities/social media
	Own provider
	National Public Radio
	Patient testimonials
Research questions to prioritize	What are COVID-19 infection outcomes in patients with IBD?
	Do medications influence outcomes of COVID-19 infection in patients with IBD?
	Do medications influence the risk of developing COVID-19 in patients with IBD?
	Will COVID-19 infection make IBD symptoms worse?
	How will IBD patients respond to COVID-19 vaccination?

Abbreviation: IBD, inflammatory bowel disease.

### Fears

#### Fears of exposure

Participants described a number of fears related to being exposed to COVID-19, particularly when accessing healthcare, both for emergent situations (eg, allergic reactions) and IBD-related care (eg, obtaining medications, infusions, screening procedures). Many participants expressed general concerns about being in public or in close proximity to providers and other patients. Some described the need to weigh risks and benefits of obtaining healthcare services or expressed the importance of protecting oneself from potential exposure.

*I felt like that when I went to go get the infusion. I’ve had to go, maybe, twice during the pandemic, and I’m just scared to go out, you know? And then, if I see anybody starting to do, like, a little cough or a clearing their throat, I get really nervous. And just as soon as they give me something or they hand me something, I’m there with the hand sanitizer. I’m wearing a face shield. It’s like, you feel like you’re going out to a battle. (FG1)*

In addition, some expressed concern about people not taking appropriate precautions in healthcare settings to prevent transmission (eg, insufficient masking).

*The doctors’ offices, at least within my neighborhood…. They’re not vigilant, at all, about protection, and they’re not vigilant—at all, about their waiting rooms. I absolutely do not feel safe going to the doctor. (FG4)*

More specifically, some participants expressed concerns about the masking at physicians’ offices as an example of lack of vigilance.

*Sometimes the staff at these places are too lax. I had to go to a regular visit at my primary, and I saw behind their glass partition, there were three or four staff members not wearing masks. (FG3)*

In addition to healthcare settings, many expressed fears of being exposed to COVID-19 through activities of everyday life, particularly as someone living with IBD and potentially having a weakened immune system. Participants described fears of being exposed to COVID-19 through friends and family, at work or school, and in public settings, including public bathrooms. Many discussed concerns that others were not taking COVID-19 seriously and felt they could not trust other’s behaviors.

*I’m a healthcare professional… And now that all this has happened, being that I am severely immunocompromised and all that, it’s like, how do I go back to work[?] [FG1]*

*It’s like you don’t trust people anymore, which is really sad. It’s like you have to kinda walk around, like, do I trust where you’ve been, who you’ve been hanging out with, or do I trust that third party that saw you?…. So it’s scary and lonely and just weird. You know, you have a lot of anxiety, like when does this end? When can I see my friends again? (FG2)*

#### Fears of being high risk

Many participants described fears related to being potentially high risk for negative outcomes related to COVID-19. In particular, many discussed concerns related to their IBD medications weakening their immune system and leaving them less equipped to fight COVID-19. Some described weighing risks and benefits of staying on their IBD medication, although some felt reassured by their providers’ encouragement to continue their treatment. Others felt reassured that certain medications (eg, steroids) have also been used to treat COVID-19.

*I take not only Remicade, but also azathioprine. So they’re two medications that affect my immune system, and that worries me a lot. I’m always thinking, like, “Okay, so is it that the more immunosuppressants that you take, is it a higher chance that, I’m gonna be really affected by the virus?”… It’s just the worry that you’re immunocompromised. Is it gonna be worse? Would I survive it? (FG1)*

Furthermore, participants described a sense of anxiety about the lack of clarity about whether or not they were high risk as someone living with IBD.

*It just became very lonely, and almost scary, too, just the fear that was instilled from news reports, conflicted news reports, being susceptible, being not; being high-risk, being not. (FG2)*

*I’m diagnosed with ulcerative colitis, and …with someone that has IBD, it’s scary to know that you might not know what else you might get besides respiratory symptoms. You don’t know if your IBD symptoms might arise. You don’t know how severe it might be or how not severe. (FG1)*

Some feared that if they contracted COVID-19, they might have a poor outcome or that their IBD could worsen.

*I had such a severe flare that, when the pandemic started, my mother and I had a discussion about the fact that we know that if we catch COVID-19, we will die. And so we’ve put in place measures for what happens then. (FG3)*

*I’m worried if I get COVID-19, and then what if it makes my symptoms worse? (FG1)*

#### Healthcare-related fears

In addition to fears related to COVID-19 exposure when accessing healthcare, many described fears related to potential lack of access or delay in receiving IBD-related treatment or medication. Participants discussed concerns about potential for IBD treatment delays as healthcare prioritized COVID-19, logistical issues in accessing medication (eg, pharmacy closures), medication cost increases, IBD medication shortages if they were used to treat COVID-19, and providers taking IBD patients off immunosuppressant drugs.

*This is my fifth biological that we’ve tried. And it’s working right now, and I’m grateful…. but I can’t afford to try a new biological currently, especially amidst a pandemic…And so it’s definitely a major fear that more research is gonna come out with some biological and be like, “Oh, no, like, this is the sure-all treatment,” and then it’s just gonna fly off the shelves, and we’re gonna be SOL. (FG4)*

While less common in the initial focus groups, a few participants also described concerns related to lack of access to or safety of COVID-19 vaccines.

*When we get to a point where there is a vaccine, it’s like, how do I get it? Will it be hard to get one? Where there be enough? Will I need my doctor to write a prescription… if they do administer them in some sort of order…is having IBD, where does that put you in the high need of needing a vaccine? (FG1)*

### Challenges

#### Challenges related to accessing healthcare

Many participants described challenges accessing IBD-related healthcare during the COVID-19 pandemic. Many discussed how it became more difficult to access healthcare providers and obtain adequate, appropriate, and timely care for their IBD during COVID-19 due to office closures, lack of in-person visits, or providers not taking on new patients.

*Living with a chronic disease for as long as I have, I’m on a certain schedule of where I would see my doctors every three months, and it was just a lot of hands-on…but then when everything shut down, and now I haven’t even been physically in to see my GI in a couple of months, that has been frustrating. (FG2)*

*I can’t get in to see a rheumatologist here. I do have also a great GP locally that does try to help all she can, but she’s just a GP so she can basically give me more steroids and like, “I’m sorry.” It’s just really tough to get in, because no one’s taking anyone. (FG3)*

They also discussed how it became more tedious and logistically challenging to obtain healthcare due to issues such as limited hours and availability of providers, COVID-19 testing requirements, or inability to bring a support person to appointments.

*Before you get any procedure done, you have to have a COVID-19 test. And I have a lot of anxiety about it getting back on time….so that adds a whole new aspect of every time you wanna do like a colonoscopy… it just adds a whole new stress. (FG2)*

In addition, some participants described challenges accessing and paying for medication due to COVID-19-related delays or closures, with some not getting medication on time and experiencing treatment delays.

*I don’t have insurance…. I’ve had to go through patient assistance programs. And just, with this whole COVID-19 thing, everything’s been set back, mail and all that stuff. So it’s been harder to get a hold of all these things that are necessary for me to receive the medication. (FG1)*

*And then there was one point where I couldn’t get my Remicade infusions on time, so I fell into this, like, depression. And like, it was really scary, and, like, I don’t wanna go through that ever again. (FG1)*

Finally, some experienced challenges navigating health insurance or felt healthcare costs increased during COVID-19.

#### Challenges navigating everyday life

Participants described a number of challenges related to navigating everyday life during the pandemic. Some noted that it was generally harder to get out of the house to do the things that brought them joy. Examples of more specific IBD-related concerns noted during the discussion included lack of access to open, noncrowded public bathrooms, concern about toilet paper shortages, and issues navigating natural disasters (eg, hurricanes) during COVID-19 as an IBD patient.

*…That’s always something that’s on my mind as an IBD patient for like my entire life, just having to be a little more critical about where I stop, or deciding if I wanna actually go out for the whole day and not know where I’m gonna have to go to the bathroom. (FG2)*

*I noticed simple things became a lot more difficult and simple things that might be more important to someone with IBD. So everybody was making jokes, at the beginning of COVID-19, about finding toilet paper. You know, that was funny to a lotta people. But I think, to us with IBD, it wasn’t funny. It was scary. How long are places gonna be out of it? Just simple things became a lot more difficult (FG1)*

#### Challenges related to relationships with others

Many participants described challenges related to their relationships with others that arose during the COVID-19 pandemic. First, participants expressed concerns about how family and friends were impacted by measures taken to protect the person living with IBD.

*Because I’m on multiple immunosuppressives, I can’t go near anyone except for my fiancé and, my grandparents because they stay quarantined… And it affects even my fiancé. He hasn’t seen any of his family because they all work. (FG3)*

*Everybody has to sort of take on extra work and take on extra precautions as well…. it’s a whole household’s effort rather than just me. And that can make me feel a bit guilty. (FG4)*

Many discussed the heavy mental load of navigating relationships and decision-making about if and how to safely see other people, with some emphasizing that they had to be more careful than those without health issues.

*[Before COVID-19] I was just all like, “Where are the bathrooms located? What kind of physical activity will we be doing?” And now it’s just like, “Are you gonna wear a mask? Who have you been with? Who have you seen? Where have you traveled?” I guess the questions have shifted gears. (FG2)*

*I think that what’s been stressful has been navigating social situations where I’m taking more precautions than other people at this point, and navigating social scenarios, like family scenarios where I want to be more cautious than others at this point. Other people might not have a heath condition or just might not be as inclined to worry about it. (FG2)*

Many described experiencing social isolation and loneliness during the pandemic due to the precautions they were taking.

*You feel kinda lame when you’re like, “I’ll still hanging out on Skype or Zoom,” and they’re all hanging out in person. You’re like, “You can call me.” So it’s kind of sad, mostly, just seeing everyone else getting back to something that’s normal. (FG1)*

*I’m a really social person, and I really miss that physical interaction and getting to know people in person. So just being at home all the time has really took a toll on my mental health, which has affected my IBD and every other symptom that I have… (FG4)*

*I’m over 65. I’m immunosuppressed. I’m in the high-risk category. I’m not gonna meet my friends and do this or do that, and I wish I could. I miss them all very, very much, and family, but, I’m restricting myself. (FG5)*

#### Challenges related to others’ perceptions of COVID-19 or IBD

Finally, many participants described feeling hurt or frustrated by others not taking COVID-19 seriously or not understanding the implications of having IBD during the pandemic. Some struggled to understand why people were not willing to take steps to protect others, like wearing a mask, and felt invalidated by their lack of concern.

*…Our immune systems are suppressed. We have to take everything much more seriously. So we take masks more seriously….it’s been emotional for me because I have been trying to understand why other people can’t do something as easy as wearing a mask for others. And it’s gotten to the point where I just break down and cry. And it’s like I literally am stuck. I’m doing everything I can on my part, but others can’t on theirs. (FG4)*

*It almost felt disrespectful when people were like, “I don’t need to wear a mask. If I get it, I get it. I’ll fight it.” …I’m trying to think how to explain it. It was hard. It was hurtful when friends and family didn’t take it seriously, even to protect me…. (FG2)*

### Information Preferences

Participants were asked about their preferences for receiving information related to COVID-19, including timing, mode of delivery, and preferred information sources.

#### Timing of information

Preferences related to timing of information varied. Some participants said they would prefer to hear new information right away, while knowing it may evolve or change.

*But, the thing is, I’m the type of person who, regardless of the situation, even if it’s not about COVID-19 and IBD, I wanna know now!….. Like, you can change your stance on it, but I’d rather be doing the thing at the start, than awaiting and then, like, shit gets bad. (FG3)*

Others preferred to wait until findings were more thoroughly researched and potentially more accurate. Some emphasized potential harms of shifting information like increased skepticisms or false hope.

*Every day, it’s an update where they’re like, “Oh, we got close to a vaccine.” And then, the next day, they’re like, “Oh, well, someone got sick.” …I feel like my hopes get high, and then they’re low again. And so I think I would rather just wait’til all the information is there instead of learning quickly. (FG1)*

Some participants said that their timing preference would depend on the trustworthiness of the source, urgency or actionability of the information, potential for harm from early information, or potential for information to change, or suggested that caveats should be included when presenting new information.

*Is both an option? I almost wanna know the information, but I want it to have, like, a big asterisk in front of it, like, “Here’s what we know now, but let’s give this a while for more comprehensive reports to come out.” So we have this information, but it needs more time, so almost kind of both, I guess, would be my ideal situation. (FG4)*

#### Mode of delivery

Preferred methods of information delivery varied, with preferences including: websites, social media, email, phone calls, texts, or electronic medical record messaging.

*Once in a while, I’ll go online and check the website, but when I get an email, I immediately check. (FG2)*

*I would trust getting it through Instagram or through Twitter—some place that I can look at it if I want, or if it’s not relevant to me, I don’t have to immediately see it. (FG4)*

At the same time, other participants expressed disliking specific information sources, including phone calls, social media, television news, or email.

*I get real nervous about social media stuff. I guess I’m an old fogie now. I was on Facebook less than a year after it started. So I’ve lived through it all, and now I’m fearful of it in some strange way. (FG4)*

Some noted the importance of using multiple modes of delivery when sharing information, and some felt that their preferred mode of information delivery depends on issues such as the importance or novelty of the information or the level of detail involved in communicating.

*I like seeing it on a trusted website as well. But if it was new information or something—like, some sorta breakthrough or something, I would want it emailed in case I don’t check for a while. (FG1)*

#### Preferred sources

Sources that participants described utilizing to gather information or as being trustworthy varied. Sources mentioned in all or nearly all focus groups included the Crohn’s & Colitis Foundation, government or intergovernmental organizations (eg, centers for disease control and prevention [CDC], world health organization, health departments), social media (patient communities or particular influencers), healthcare providers, and research entities (organizations, news, journals/articles, or scientists). Friends and family and news organizations were also noted as sources of information by some participants.

*Crohn’s & Colitis Foundation, and those kinds of places, they want what’s best for IBD patients, in my opinion. They’re not making a buck off of, like, “Oh, here’s the cure for COVID-19.” You know? (FG3)*

*There was a website…. And I think the CDC was part of it, and I just remember it was a very big white website with a lot of information on it. And I was trusting that because it was ran by multiple international government agencies. (FG4)*

*Reddit has been kind of a decent source for Crohn’s and COVID-19. (FG4)*

*It really depends on what the exact information is, but I feel like, if I did hear that my GI would like cosign it and approve it and get behind it, I would trust it more. So that would probably be the first place I check personally (FG2)*

Several participants described the importance of being able to verify information, such as seeing information through multiple news sources, or having links/citations in articles.

*If I see the same information given out on various sources, then it looks like it’s a real thing. They’re not giving out fake remedies and silly things like that. (FG3)*

### Research Questions

Most of the research questions highlighted by focus group participants related to experiences, susceptibility, and risk of COVID-19 among people living with IBD. Participants wanted to know if people with IBD or taking specific IBD medications were at a higher risk of contracting COVID-19 or having a poor outcome, and desired more information about outcomes experienced by those who had contracted COVID-19.

*I’d be curious to know outcomes for patients that have been infected with COVID-19 who have the comorbidity of an IBD. If there’s a higher incidence of ICU hospitalization versus people who are able to manage at home versus heaven forbid, death—that kinda stuff. (FG4)*

*Is there an additional risk because of IBD?… And, you know, like obesity or high blood pressure, diabetes, they mention that as high risk for COVID-19, but they don’t mention IBD. (FG5)*

Additional research questions mentioned were related to long-term effects of COVID-19 or the COVID-19 vaccine among IBD patients, issues related to stress and IBD flare-ups, and how a post-pandemic world might look for people living with IBD (eg, whether clinical trials will change, or whether working from home might become the norm).

### Member Checking Focus Groups

Participants in both member checking focus groups validated the fears and challenges presented from the initial focus groups as generally reflecting their perceptions and experiences earlier in the pandemic. The amount that these fears and challenges have decreased over time varied by individual concern and by participant. When discussing the COVID-19 vaccines, participants in these focus groups noted some additional research questions (eg, how IBD patients will respond to the vaccine) and additional concerns (eg, potential for interaction with IBD medication, feeling forgotten in the vaccine distribution).

*I’m excited and, I guess, frustrated….my mom has IBD too, or Crohn’s, I should say. I feel like we almost got forgotten about in terms of priority. (MC FG2)*

Like the previous focus groups, information preferences varied. New suggestions for sharing information that emerged from the member checking focus groups included developing an app for sharing IBD news and personal health tracking, offering information geared toward children living with IBD during the pandemic, and sharing testimonials about IBD patients’ experiences getting the COVID-19 vaccine.

*….Especially people who are not from a research science background, testimonials are huge. Putting a face or a story to someone’s experience can go a long way as opposed to a number. (MC FG1)*

## Discussion

To our knowledge, this is the first qualitative study of patients with IBD focusing upon impacts of the COVID-19 pandemic on the IBD community. A prior Scandinavian study used surveys in an outpatient IBD clinic to assess concerns of patients with IBD during the pandemic. This study focused upon mitigation techniques such as quarantine and avoiding in-person visits.^8^ No data were obtained surrounding patient prioritization of research questions based on their fears. A second web-based survey on disease-related experiences of patients with IBD and concerns during the COVID-19 pandemic showed that 62% feared an increased risk of severe COVID-19. Many canceled appointments and others (up to 7%) reduced or paused medications because of these fears.^9^ Our findings highlight the importance of the patient voice in prioritizing a research agenda and utilizing trustworthy mechanisms for dissemination of those findings. By utilizing focus groups with an open-ended guide, we were able to elicit broad themes of importance to patients with IBD. The prioritized research questions identified by our focus groups include an emphasis on IBD (and IBD medications) and how IBD or its treatment may impact COVID-19 infection and COVID-19 vaccination outcomes. Particularly as time advanced, another important focus identified by IBD patients was further information surrounding COVID-19 vaccination in IBD populations. Our focus groups can help inform future communication practices and methods for delivering information to patients. As preferred modes of communication varied among our participants, a multimodal approach (email, websites, social media) may be an effective strategy. Some participants suggested a preference for direct emails with important information. Additionally, some participants spoke to the importance of patient testimonials and learning from other patients’ stories. Emphasizing testimonials in communication may help to relay important messages to our patient community.

Our findings of prioritized research questions from patients do mirror those of the IBD research community. Early in the pandemic, investigators developed a method to better report and understand COVID-19 complications in patients with IBD. The SECURE-IBD registry of 1439 cases of COVID-19 in IBD patients from 47 countries has demonstrated that thiopurine therapy (aOR 4.08, 95% CI 1.73–9.61) and combination therapy with anti-TNF and thiopurine (aOR 4.01, 95% CI 1.65–9.78) were associated with increased risks of severe COVID-19 outcomes.^6^ This registry is ongoing and continuing to collect important information on outcomes of COVID-19 specific to IBD. Patient fears of accessing healthcare likely impacted their abilities to continue routine disease management. There was a push to digital healthcare technology to manage patients with IBD remotely,^10^ but not all could access this method of continued care. Procedures such as colonoscopies were delayed for many indications (including IBD)^11,12^ and medical therapies such as infusions were often postponed,^12^ impacting disease control. These issues were reflected in our focus groups, as participants discussed fears and challenges related to accessing IBD-related healthcare during the pandemic. Additionally, participants spoke of vigilance, and episodes where they saw lapses in healthcare offices. Robust adherence to masking, hand washing, and social distancing (by staff and patients) in office can help provide reassurance to patients. As vaccinations have become available, prioritized research questions for IBD patients have evolved, with a focus on COVID-19 vaccination outcomes in IBD patients. There are a number of studies addressing vaccination in IBD patients,^13,14^ although further long-term data are needed. Specifically, the Prevent COVID (www.ibdpartners.org/preventcovid) study was developed in response to this patient-prioritized research agenda, and communicates results to patients in a real-time fashion using some of the information preferences identified in this qualitative study.^15^

There are a number of strengths to this qualitative study, including the broad recruitment via electronic means such as social media. We utilized a virtual format for the focus groups, and thus were better able to include geographically diverse participants. There are also a number of limitations to this study, in that computer access was required for participation. This likely limited individual participants to those of a higher socioeconomic class. There is also the potential for selection bias, as people with greater concerns surrounding COVID-19 may have been more likely to participate. As there was no in-person communication or interaction, the virtual format may also impact the back and forth communication between participants. However, transcripts did demonstrate ample contribution by each individual to the discussion and levels of concern did vary among participants. While we recruited broadly and emphasized diversity in age, gender, and ethnicity in our recruitment materials, we did not capture demographic data of participants to confirm specific findings by gender or age. Additionally, while we had adequate sample size to reach saturation in our first round of focus groups,^16^ we had limited numbers of focus groups for member checking. Therefore, we cannot be certain that we reached saturation on new themes in the second round. Our focus groups included individuals with self-reported IBD, no validation of medical records was performed. However, similar recruitment methods for a Crohn’s and Colitis Foundation cohort, IBD Partners, have demonstrated >97% of patients included have IBD on medical record validation.^17^ Thus, our findings should be applicable to the IBD population.

In summary, our findings demonstrate that patient prioritization of information needs via virtual focus groups is feasible and can readily inform an IBD research agenda moving forward. A better understanding of a patient-prioritized research agenda, identified trustworthy information sources for patients, preferred methods of communication, and the prioritization of accurate information will inform the COVID-19 IBD research agenda in the future.

## Data Availability

Data for this manuscript are not publically available.
